# Molecular and Biochemical Characterization of Xylanase Produced by *Streptomyces viridodiastaticus* MS9, a Newly Isolated Soil Bacterium

**DOI:** 10.4014/jmb.2309.09029

**Published:** 2023-11-17

**Authors:** Jong-Hee Kim, Won-Jae Chi

**Affiliations:** 1 Department of Food and Nutrition, Seoil University, Seoul 02192, Republic of Korea; 2 Species Diversity Research Division, National Institute of Biological Resources, Incheon 22689, Republic of Korea

**Keywords:** Xylanase, *Streptomyces viridodiastaticus*, GHF93985.1, endo-(1,4)-β-xylanase

## Abstract

A xylan-degrading bacterial strain, MS9, was recently isolated from soil samples collected in Namhae, Gyeongsangnam-do, Republic of Korea. This strain was identified as a variant of *Streptomyces viridodiastaticus* NBRC13106^T^ based on 16S rRNA gene sequencing, DNA–DNA hybridization analysis, and other chemotaxonomic characteristics, and was named *S. viridodiastaticus* MS9 (=KCTC29014= DSM42055). In this study, we aimed to investigate the molecular and biochemical characteristics of a xylanase (XynC_vir_) identified from *S. viridodiastaticus* MS9. XynC_vir_ (molecular weight ≈ 21 kDa) was purified from a modified Luria–Bertani medium, in which cell growth and xylanase production considerably increased after addition of xylan. Thin layer chromatography of xylan-hydrolysate showed that XynC_vir_ is an endo-(1,4)-β-xylanase that degrades xylan into a series of xylooligosaccharides, ultimately converting it to xylobiose. The *K*_m_ and *V*_max_ values of XynC_vir_ for beechwood xylan were 1.13 mg/ml and 270.3 U/mg, respectively. Only one protein (GHF93985.1, 242 amino acids) containing an amino acid sequence identical to the amino-terminal sequence of XynC_vir_ was identified in the genome of *S. viridodiastaticus*. GHF93985.1 with the twin-arginine translocation signal peptide is cleaved between Ala-50 and Ala-51 to form the mature protein (21.1 kDa; 192 amino acids), which has the same amino-terminal sequence (ATTITTNQT) and molecular weight as XynC_vir_, indicating GHF93985.1 corresponds to XynC_vir_. Since none of the 100 open reading frames most homologous to GHF93985.1 listed in GenBank have been identified for their biochemical functions, our findings greatly contribute to the understanding of their biochemical characteristics.

## Introduction

Xylan is a major component of hemicellulose, which constitutes plant cell walls, and is the third most abundant biopolymer. It is a heterogeneous polysaccharide in which D-xylose forms branches with acetyl, arabinosyl, and/or methyl-glucuronosyl residues by β-1,4-linkages. Among the known xylan degrading enzymes, endo-(1,4)-β-xylanases (E.C. 3.2.1.8) cleave β-1,4-glycosidic bonds between D-xylose residues in the main chain, forming small oligosaccharides [3]. Xylanase is used in various industries, including the pre-bleaching process of pulp to remove hemicellulose bound to cellulose, animal feed to improve digestibility, food and bakery industries (xylan content affects dough hardness), fruit and vegetable processing, ethanol fermentation, and xylitol production [3, 11]. Xylooligosaccharides prepared from xylan by xylanase treatment have potential prebiotic properties and can be used as “functional foods” [6].

Owing to its various industrial applications, xylanases have been isolated and identified from several fungi and bacteria [28]. In particular, production from microorganisms has the advantage of utilizing rapid microbial growth and well-established fermentation technologies. Thus, many attempts have been made to isolate new microbial strains as sources of novel enzymes with useful and distinct characteristics [5, 15, 20].

*Streptomyces* is a Gram-positive bacterium that abundantly inhabits the soil. It is an industrially useful microbe that produces various secondary metabolites used in the production of antibiotics, anticancer drugs, immuno-suppressants, and other biologically active substances [14]. In addition, *Streptomyces* can produce and secrete useful hydrolytic enzymes, such as proteases, amylases, and pectinases, that hydrolyze organic matter in the soil [7, 13, 37].

In our previous study, we isolated and identified xylan-degrading microorganisms from soil samples. The strain MS9, which showed highest xylanase activity, was identified as a variant of *Streptomyces viridodiastaticus* NBRC13106^T^. In this study, we aimed to investigate the molecular and biochemical characteristics of a xylanase, which was first identified from *S. viridodiastaticus*.

## Materials and Methods

### Chemicals

All chemicals were purchased from Sigma-Aldrich (USA), unless otherwise noted. Luria–Bertani (LB) medium was purchased from BD Difco (USA) and protein markers were obtained from Amersham Pharmacia Biotech (UK). Polymerase chain reaction (PCR) kits and DNA-modifying enzymes were purchased from TaKaRa Bio (Japan).

### Isolation of Microbial Strain

Soil samples were collected from Namhae City, Gyeongsangnam-do, Republic of Korea. Serially diluted solution of the sample in distilled water was spread on an LB agar plate containing 0.2% xylan azure, and incubated at 40°C for 2 days. The culture temperature was set at 40°C to isolate strains capable of producing thermophilic enzymes as described [8]. Strains that could hydrolyze xylan azure were identified by a color change around the colony, selectively transferred to new LB agar medium, and cultured under the same conditions. The strain with the highest xylanase activity was named MS9 and used in this study. For 16S rRNA coding gene analysis, a genomic DNA extracted from strain MS9 (template) and bacterial universal primers [4], 27F (5'-AGAGTTTGATCCTGGCTCAG- 3') and 1492R (5'-GGTTACCTTGTTACGACTT-3'), were used to perform PCR, as described previously [31]. The 16S rRNA gene sequence of the MS9 strain was obtained using Basic Local Alignment Search Tool (BLAST) program [1] at NCBI, and was submitted to the GenBank database; gene sequences of the related strains were collected from the EzTaxon server [9]. Multiple alignments with the nucleotide sequences of related *Streptomyces* were performed using ClustalW software [30]. A phylogenetic tree was constructed using the neighbor-joining method [26] in the Mega6 program [29]. The evolutionary distances were calculated using the Poisson correction method [38].

### DNA–DNA Hybridization

Genomic DNAs from strain MS9, *S. viridodiastaticus* NBRC13106^T^, and *S. atrovirens* NRRL B-16357^T^ were prepared from cells grown on LB plates using a Genomic DNA Extraction Kit (DyneBio Inc., Republic of Korea). *E. coli* KCCM12119^T^ was used as the negative control. Probe DNA preparation and hybridization reactions were performed using the DIG High Prime DNA Labeling and Detection Starter Kit II (Roche Applied Science, Germany), according to the manufacturer’s protocols. The resulting hybridization signals were measured using Quantity One Program (Bio-Rad Laboratories, Inc., USA). The signal from strain MS9 was considered to be 100%.

### Phenotypic and Biochemical Characteristics of Strain MS9

Biochemical characteristics were observed using API 20NE and API ZYM strips (Biomérieux, France), according to the manufacturer’s instructions, except that the bacterial suspension was prepared in AUX medium supplemented with 1.0% (w/v) NaCl and a trace element solution. Growths at different pH values (4.0–11.0 at intervals of pH 1), various concentration of NaCl (0–10% at intervals of 1%), and different temperatures (20–52°C) were determined using LB agar plate.

### Cell Growth and Xylanase Production

The strain MS9 was inoculated into LB broth or LB broth supplemented with 0.3% xylan (LBX medium) and cultured at 40°C for 4 days with vigorous stirring. The culture medium was collected at regular intervals, and the cell density was measured at 600 nm (OD_600_). After centrifuging the sample at 10,000 ×*g* for 20 min, the cell-free culture broth was collected to measure xylanase activity using the dinitrosalicylic acid (DNS) method [23].

### Determination of Xylanase Activity by DNS Method

The enzyme solution (100 μl) was added to 4.9 ml of 20 mM Tris-Cl (pH 7.0) containing 0.5% (w/v) beechwood xylan substrate and incubated at 60°C for 10 min. DNS reagent (5 ml), containing 6.5 g DNS, 325 ml of 2 M NaOH, and 45 ml of glycerol in 1 L distilled water, was mixed with the reaction solution and heated in boiling water for 10 min. After cooling in an ice-water bath, the absorbance at 540 nm (A_540_) was measured using a Spectronic Unicam Genesys 8 spectrophotometer (Thermo Fisher Scientific, USA). 1 unit (U) of xylanase was defined as the amount of enzyme producing 1 μmol of xylose per min under the above assay conditions. D-Xylose was used as a reducing sugar reference to prepare a standard curve.

### Purification of a Xylanase from Culture Broth of Strain MS9

Strain MS9 was inoculated into LBX broth and cultured at 40°C for 1 day. The cell-free supernatant was obtained by centrifuging the culture broth at 10,000 ×*g* for 20 min and (NH_4_)_2_SO_4_ was added at a final concentration of 70% (w/v). After 2 h, the protein precipitate was obtained by centrifugation (20,000 ×*g*, 60 min), which was resuspended and dialyzed in buffer-A (10 mM Tris-Cl, pH 8.0) for 12 h at 4°C. The dialysate was concentrated with a 3 kDa cut-off ultrafiltration membrane (Pall Corp., USA), centrifuged (20,000 ×*g*, 30 min) to remove aggregated proteins, and the soluble protein samples were loaded onto a Resource-Q column mounted on an ÄKTA-FPLC system (GE Healthcare Life Sciences, USA). After washing the column with buffer-A, protein elution was performed using a 60 column volume of linear gradient from 0 to 1 M NaCl in buffer-A, at a flow rate of 1 ml/min. Fractions showing xylanase activity by the DNS method were analyzed using 0.1% sodium dodecyl sulfate–15% polyacrylamide gel electrophoresis (SDS-PAGE), as described by Laemmli [19]. For the zymography of xylanase activity, a gel was prepared by adding 0.5% (w/v) beechwood xylan as a substrate, and the protein samples were directly loaded onto the gel without heat treatment. After electrophoresis, the gel was immersed in 2.5% (w/v) Triton X-100 solution and buffer B (20 mM Tris-Cl, pH 8.0) for 30 min. The gel was incubated in buffer B at 40°C for 3 h for enzyme reaction and stained with 2% (w/v) Congo red solution. For amino-terminal amino acid sequencing, the purified protein was electrophoretically transferred onto a polyvinylidene difluoride membrane (Merck Millipore, Germany) after SDS-PAGE, and analyzed by Edman degradation.

### Enzymatic Characteristics

Unless otherwise specified, the xylanase enzyme reaction was performed by dissolving 0.5% (w/v) beechwood xylan (substrate) in 20 mM Tris-Cl (pH 7.0) buffer, and the reaction was performed at 60°C for 10 min (standard condition). The effect of pH on xylanase activity was investigated using 20 mM MOPS buffer (pH 6.0, 7.0) and 20 mM Tris-Cl buffer (pH 6.0, 7.0, 8.0). The effect of reaction temperature on enzyme activity was carried out at a temperature ranging from 40 to 70°C. For the thermal stability of xylanase, the enzyme was heat-treated at 60, 65, and 70°C for 10, 20, 30, 60, and 120 min, respectively, and the residual enzyme activity was measured. The effects of CoCl_2_, MnCl_2_, MgCl_2_, CaCl_2_, ZnCl_2_, KCl, NaCl metal ions, and EDTA on xylanase activity were investigated by adding each chemical to the reaction mixture at a final concentration of 5 mM. Substrate specificity was measured for different substrates (0.5%, w/v) such as birchwood xylan, beechwood xylan, oat spelt xylan, carboxymethyl (CM)-cellulose, and Avicel.

### Thin-Layer Chromatography (TLC) Analysis

The enzyme reaction (total reaction volume, 80 μl) containing 0.5% (w/v) beechwood xylan in 20 mM Tris-Cl (pH 7.0) was performed at 60°C for 24 h. 7 μl of the reaction mixture was aliquoted and spotted on a silica gel 60 plate (Merck Millipore). The samples were double-ascended using a solvent system of n-butanol : acetic acid : water (2:1:2, v/v/v). After heating to 120°C, 10% (w/v) sulfuric acid in ethanol was sprayed on the plate to visualize the hydrolyzed spots.

### Kinetic Analysis

The Michaelis constant (*K*_m_) and maximum velocity (*V*_max_) of xylanase (2.9 μg) for beechwood xylan substrate (1.0 ~ 30.0 mg/ml) were calculated by Lineweaver-Burk plot of the initial velocity data obtained under standard conditions [22].

## Results

### 16S rRNA Gene Sequence and Phylogenetic Analysis

**Fig. 1:**
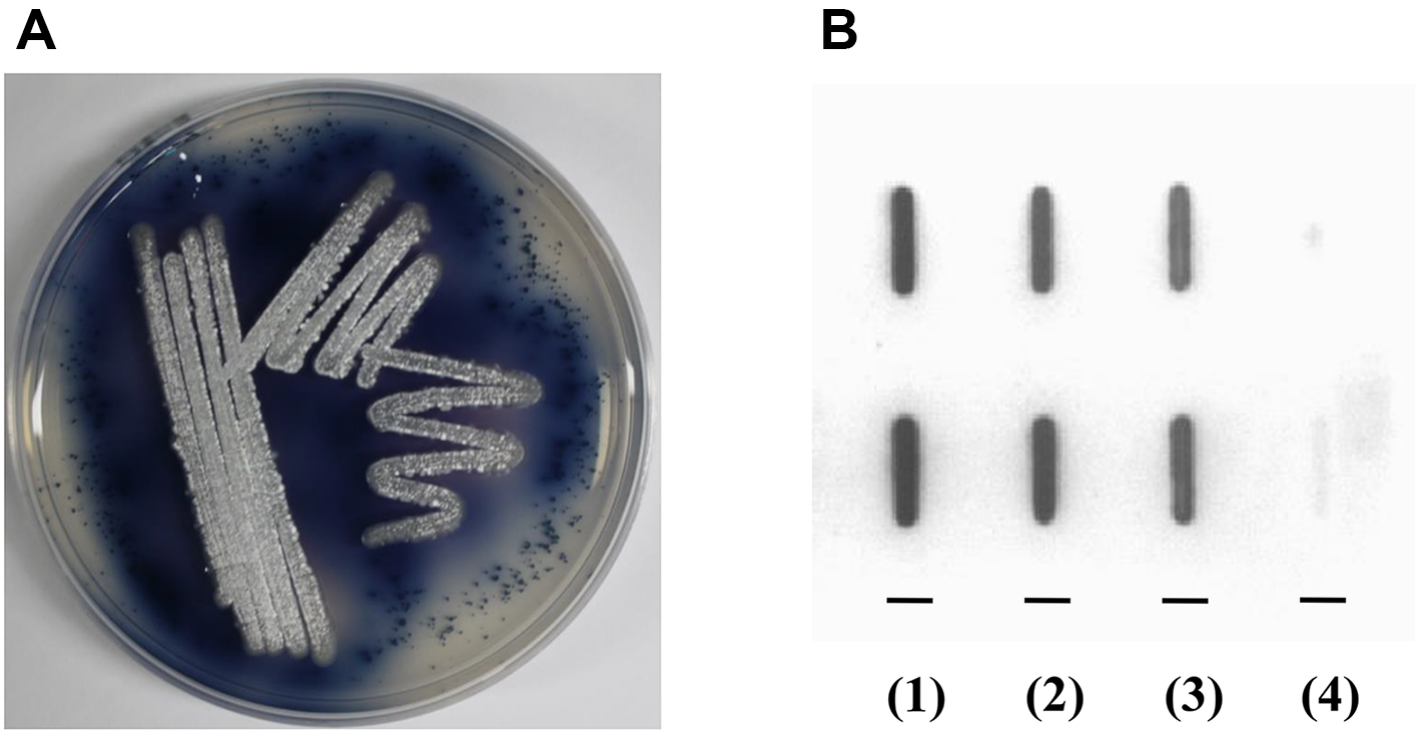
Characteristics of strain MS9. (**A**) Xylanase activity on agar plate: strain MS9 was cultured on an LB plate containing 0.2% xylan azure at 40°C for 4 days. The blue color around colonies of MS9 indicates that xylan azure was hydrolyzed by xylanase produced by the strain. (**B**) DNA–DNA hybridization analysis: (1) strain MS9, (2) *S. viridodiastaticus* NBRC13106^T^, (3) *S. atrovirens* NRRL B-16357^T^, (4) *E. coli* KCCM12119 (negative control). The signal from strain MS9 was considered to be 100%. The upper and lower panels show results of repeating the same experiment.

Strain MS9 was isolated from a soil sample collected in Namhae City, Gyeongsangnam-do, Republic of Korea (Fig. 1A). The isolate was deposited as KCTC29014 and DSM42055 in Korean Collection for Type Culture (KCTC) and Deutsche Sammlung von Mikroorganismen und Zelkulturen GmbH (DSMZ), respectively. The nearly complete 16S rRNA gene (1,421 bp) of strain MS9 was determined and registered as JN578485 in GenBank. The 16S rRNA gene sequence analysis of strain MS9 was aligned by comparison with available sequences from ExTaxon Server 2.1, which revealed that the 16S rRNA gene sequence of strain MS9 showed high similarity to the strains, *S. viridodiastaticus* NBRC13106^T^ (100%) that is considered as later heterotypic synonyms of *S. albogriseolus* NRRL B-1305^T^ [18], *S. longispororuber* NBRC13488^T^ (99.5%), *S. coeruleorubidus* NBRC12844^T^ (99.3%), *S. atrovirens* NRRL B-16357^T^ (99.2%), and *S. griseorubens* NBRC12780^T^ (99.2%). In the neighbor-joining phylogenetic tree, strain MS9 formed a subcluster (bootstrap value of 100%) with the genetically closest *S. viridodiastaticus* NBRC 13106^T^ (data not shown).

### DNA–DNA Hybridization and Chemotaxonomic Analysis

In the DNA relatedness analysis, strain MS9 showed > 90% and 70.1% relatedness to *S. viridodiastaticus* NBRC13106^T^ and *S. atrovirens* NRRL B-16357^T^, respectively (Fig. 1B), indicating that MS9 is the same species as the former, as proposed by Van Trappen *et al* [32]. MS9 strain showed good growth at pH of 5.5-10.0, NaCl concentration of 0-9% (w/v), and cultivation temperature of 20-50°C. Gray-colored spores are routinely observed on the surface of white-colored aerial mycelia on agar plates, such as ISP-2, ISP-4, YMPD, and R2YE plates [16]. Strain MS9 formed a bald phenotype and white-colored aerial mycelia on LB and LBX, respectively. Among various chemotaxonomic characteristics tested, MS9 showed a difference in that it was urease-negative and *S. viridodiastaticus* NBRC13106^T^ was urease-positive. Based on its genetic and chemotaxonomic characteristics, the strain MS9 was named *S. viridodiastaticus* MS9.

### Cell Growth and Xylanase Production of *S. viridodiastaticus* MS9

**Fig. 2:**
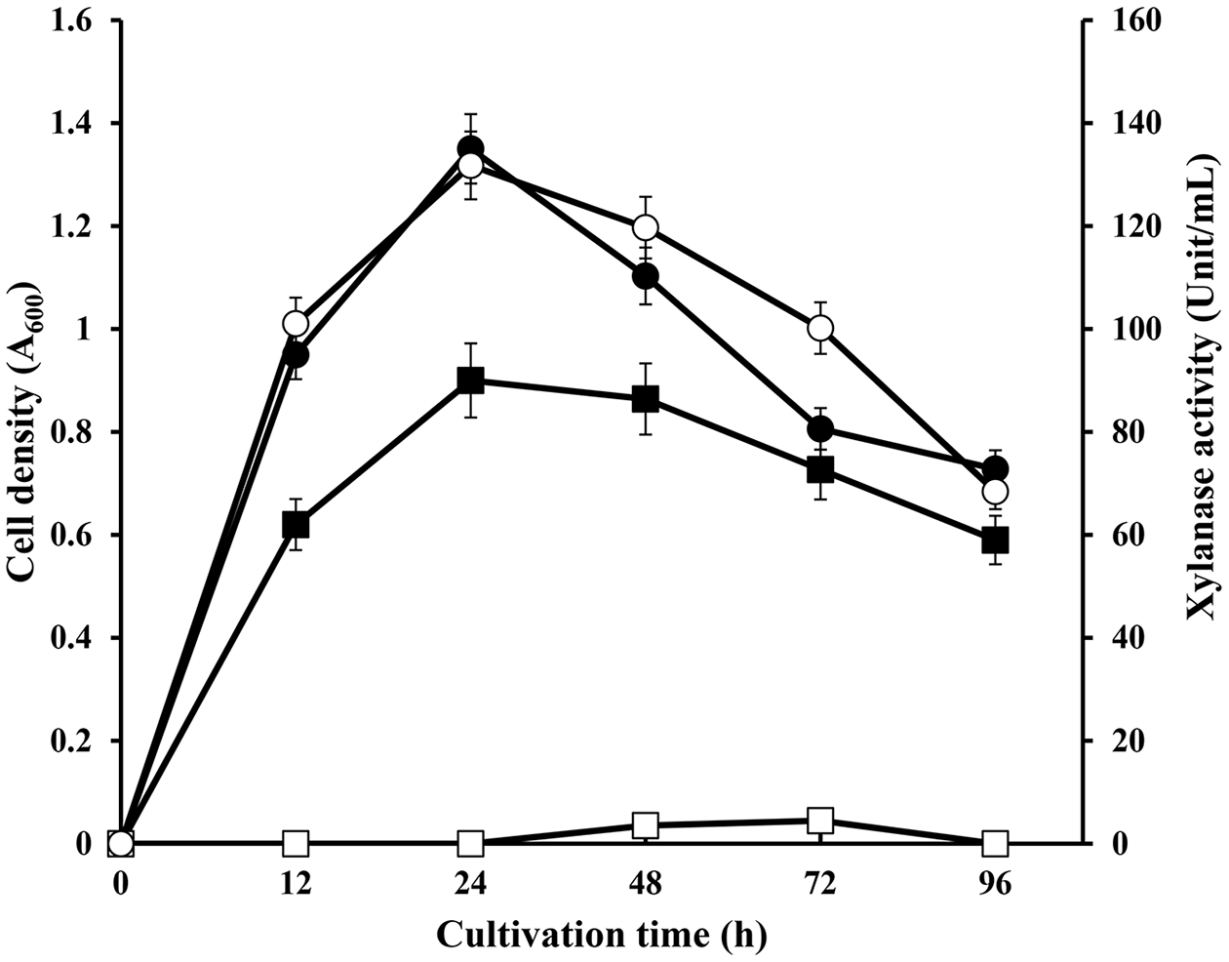
Cell growth and xylanase production in *S. viridodiastaticus* MS9. MS9 was cultured in LB or LBX broth (LB broth supplemented with 0.3% (w/v) xylan as a carbon source) at 40°C with vigorous shaking. Cell growth was measured at 600 nm, and xylanase activity was measured using the DNS method at 540 nm. ■, cell growth in LB; □, xylanase activity in LB; ●, cell growth in LBX; ○, xylanase activity in LBX.

When *S. viridodiastaticus* MS9 was cultured in LBX broth at 40°C, the highest cell growth and maximum xylanase activity were observed at 24 h cultivation (Fig. 2). When strain MS9 was cultured in LB medium, cell growth increased exponentially and reached maximum growth at 24 h of culture; however, the value was only approximately 67% of the maximum cell density when cultured in LBX. In comparison, xylanase activity showed a large difference between the two culture media, with the maximal activity in LBX medium (131.8 U/ml) being 30 times higher than that in LB medium (4.4 U/ml). This result indicated that xylanase production was induced by adding xylan to the medium, and cell growth was increased by xylan decomposition using xylanase as a carbon source.

### Purification of Xylanase from *S. viridodiastaticus* MS9

**Fig. 3:**
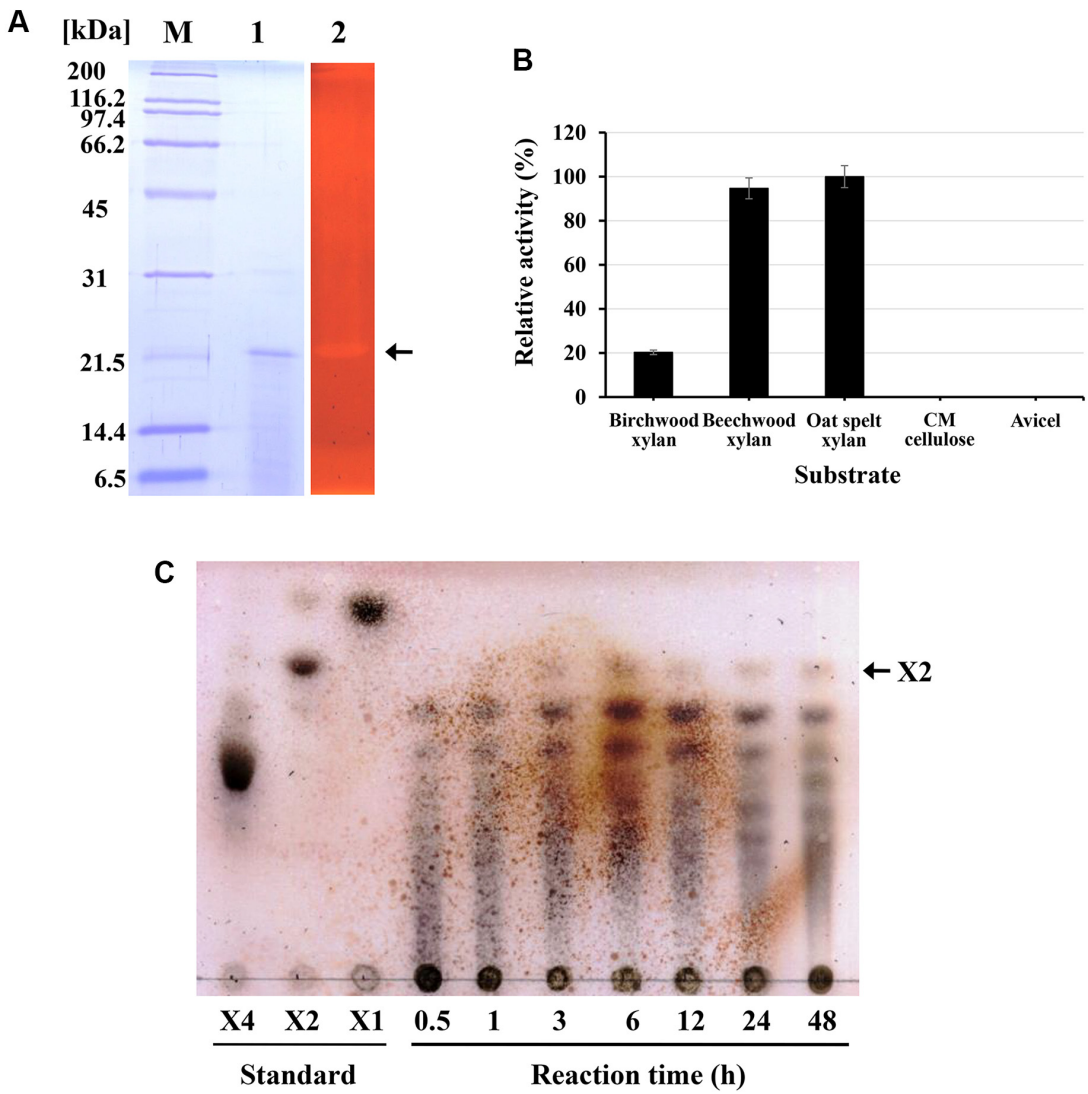
Purification and characterization of the xylanase obtained from *S. viridodiastaticus* MS9. (**A**) Estimation of molecular weight and xylanase activity of the purified protein. The xylanase-active fraction, obtained from Resource-Q column chromatography through several stages of purification, was analyzed using 0.1% sodium dodecyl sulfate– 15% polyacrylamide gel electrophoresis (lane 1). The xylanase activity of the isolated protein (molecular weight ≈ 21 *kDa*) was confirmed by a zymogram, as indicated by an arrow (lane 2). M, molecular-weight standard. (**B**) Substrate specificity was measured using the DNS method on different substrates (0.5%, w/v), such as birchwood xylan, beechwood xylan, oat spelt xylan, carboxymethyl (CM)-cellulose, and avicel. The enzyme reaction was performed in 20 mM Tris-Cl (pH 7.0) buffer at 60°C for 10 min. The enzyme activity of oat-spelt xylan was considered to be 100%. (**C**) TLC of the xylan hydrolysate obtained using purified xylanase. The enzyme reaction was carried out at 60°C for 12 h in 20 mM Tris-Cl (pH 7.0) containing 0.2% (w/v) beechwood xylan and then separated on a Silica Gel 60 TLC plate. X1: xylose; X2: xylobiose; X4: xylotetraose. The spot corresponding to X2 is indicated by arrows.

Xylanase was purified to homogeneity from the culture broth of MS9 by several purification steps, including resource-Q column chromatography. The xylanase-active fraction (fraction 4) from Resource-Q column chromatography contained a single protein with an apparent molecular weight of 21 kDa on SDS-PAGE (Fig. 3A, lane 1), which coincided with the xylanase-active band on zymography (Fig. 3A, lane 2). Purified xylanase showed high activity on xylan substrates such as birchwood xylan, beechwood xylan, and oat-spelled xylan, but no activity was detected on cellulose substrates such as CM-cellulose and avicel (Fig. 3B). These results strongly indicated that the purified xylanase was cellulase-free.

### TLC Analysis of the Xylan-Hydrolysate by Xylanase of *S. viridodiastaticus* MS9

The beechwood xylan hydrolysate produced by the purified xylanase was analyzed by TLC using a silica gel 60 plate (Fig. 3C). Various lengths of xylooligosaccharides (>xylobiose) were detected at initial step of reaction and then a final product corresponding to xylobiose was followed by further reaction, indicating that the purified protein was an endo-(1,4)-β-xylanase that can hydrolyze xylan to xylobiose. The fact that the ratio of oligosaccharides containing xylobiose does not change depending on the reaction time after 3 h of reaction also supports that the xylanase is a typical endotype hydrolase that does not recognize short oligosaccharides as substrates.

### Enzymatic Characteristics of Xylanase of *S. viridodiastaticus* MS9

**Fig. 4:**
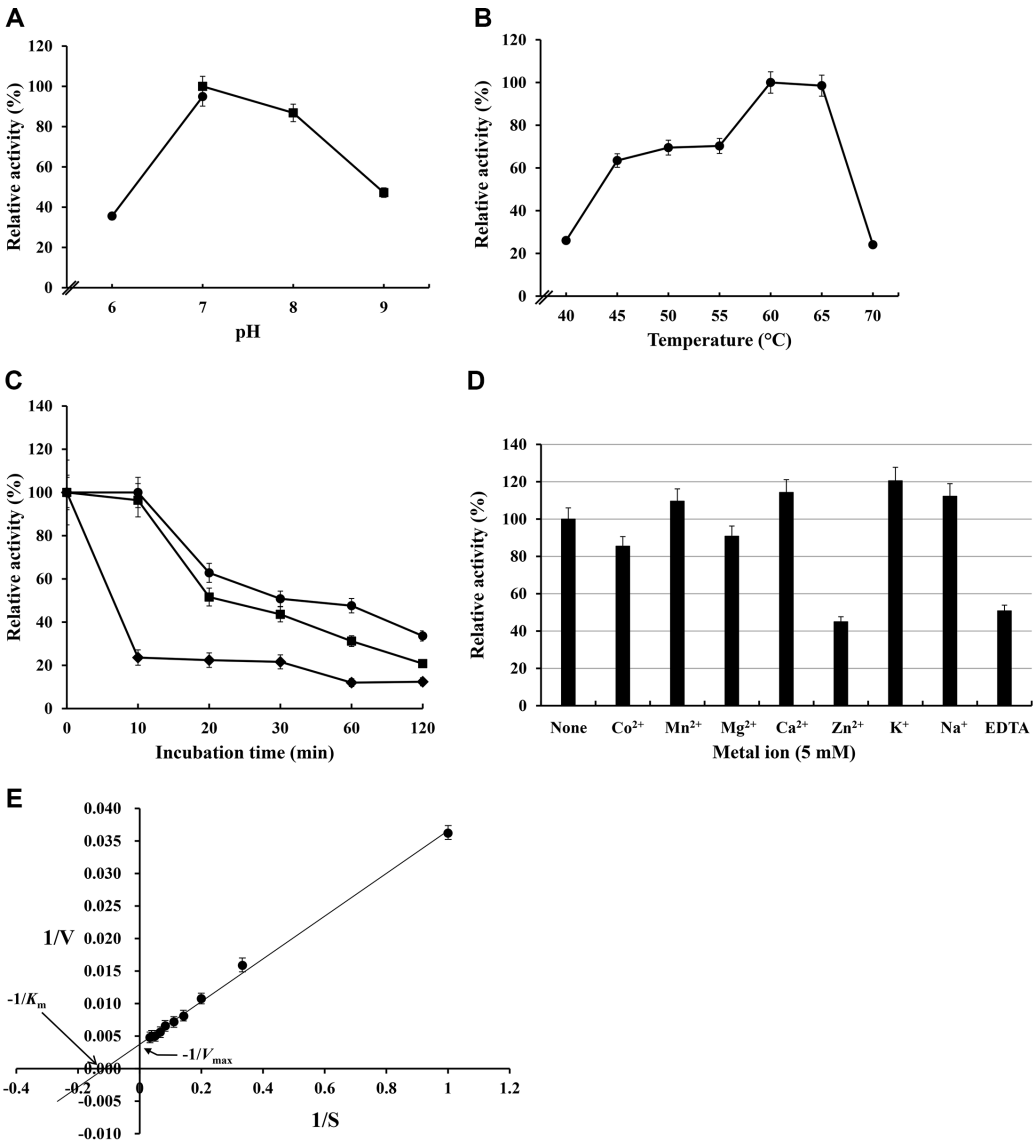
Biochemical characteristics of the purified xylanase from *S. viridodiastaticus* MS9. The following enzymatic reaction was performed at 60°C for 10 min in 20 mM Tris-Cl (pH 7.0) buffer using beechwood xylan as a substrate unless otherwise specified. (**A**) Effect of pH. The xylanase assay was performed under different pH conditions. Values obtained with 20 mM Tris-Cl (pH 7.0) were considered 100%. ●, 20 mM MOPS buffer; ■, 20 mM Tris-Cl buffer. (**B**) Effects of temperature. The xylanase assay was carried out at different temperatures ranging from 40°C to 70°C. The values obtained at 60°C were taken to be 100%. (**C**) Temperature stability. The enzyme was heat-treated at the indicated temperatures for 10, 20, 30, 60, and 120 min, and residual enzyme activity was measured. The activity value without preincubation was set to 100%. ●, 60°C; ■, 65°C; ♦, 70°C. (**D**) The effects of metal ions and chelating reagents. Each compound was added to the reaction mixture at a final concentration of 5 mM. The enzyme activity in the absence of chemicals was 100%. (**E**) Determination of kinetic parameters. Lineweaver-Burke plots were used to determine the kinetic parameters of the purified xylanase acting on beechwood xylan. Data are shown as mean values of at least three replicate experiments.

The enzyme showed high activity over a pH range of 7.0–9.0, with an optimal pH of 7.0 (Fig. 4A). Enzyme activity at pH 7.0 showed an optimum temperature of 60-65°C and significantly decreased at 70°C (Fig. 4B). The half-lives of the purified xylanase at 60 and 65°C were 20 and 30 min, respectively, however, at 70°C, it rapidly inactivated to about 20% of its initial activity within 10 min (Fig. 4C). Enzyme activity was slightly increased by Ca^2+^ (114.3%), K^+^ (120.5%), and Na^+^ (112.2%), but was inhibited by Zn^2+^ (44.9%) and EDTA (50.8%) (Fig. 4D). According to the Lineweaver-Burk plot, the *K*_m_ value and *V*_max_ of purified xylanase for beechwood xylan were found to be 1.13 mg/ml and 270.3 U/mg, respectively (Fig. 4E).

### Identification of Xylanase-Encoding Gene by in silico Analysis

**Fig. 5:**
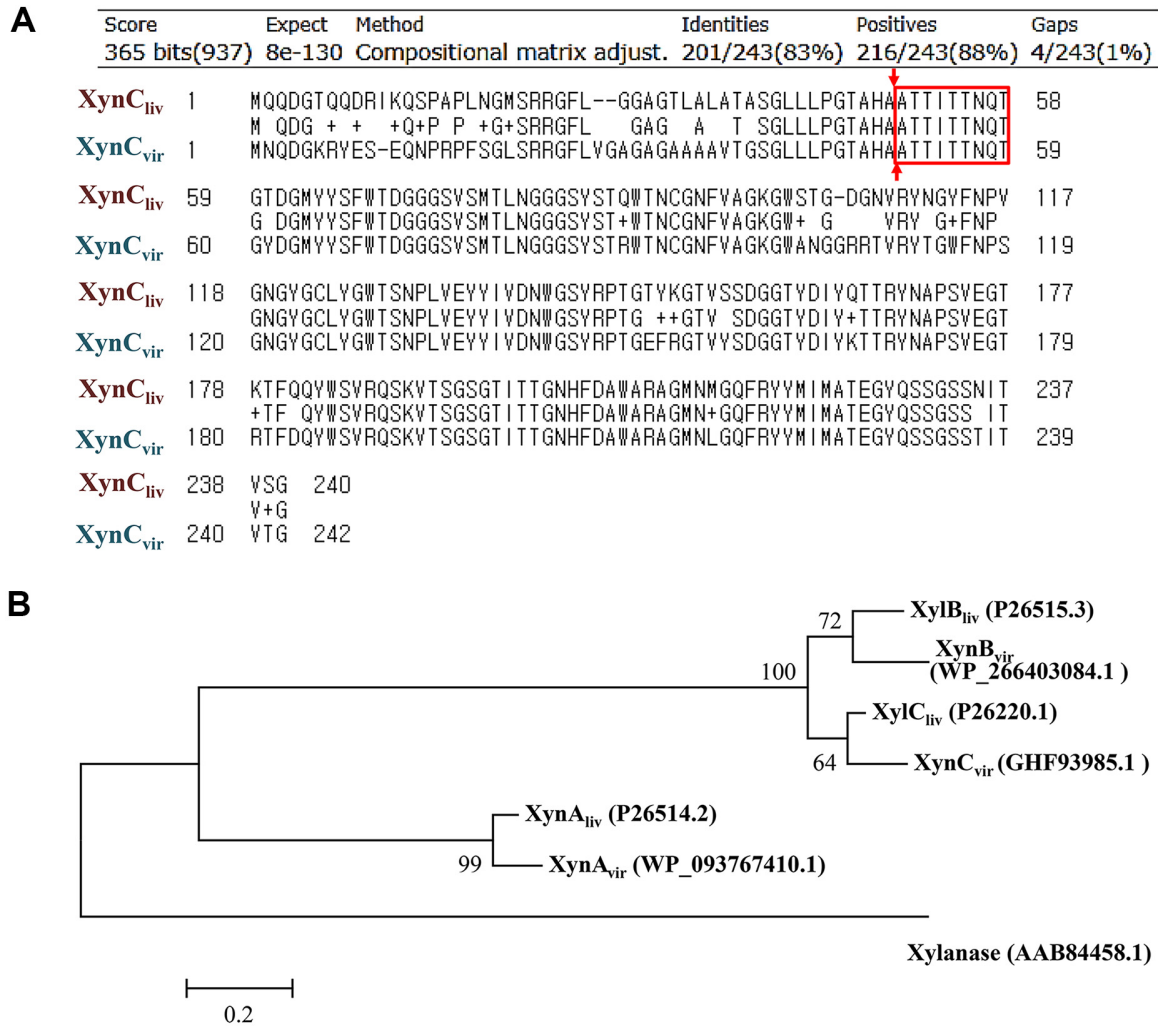
Comparison of xylanases in *S. viridodiastaticus* and *S. lividans*. (**A**) Comparison of primary structures in xylanase C (XynC_liv_) obtained from *S. lividans* and its orthologue (XynC_vir_) obtained from *S. viridodiastaticus*. The signal peptide cleavage sites predicted by SignalP 6.0 are indicated by arrows, and the amino-terminal amino acid sequence confirmed in mature form of XynC_vir_ is indicated by a box. (**B**) Phylogenetic relationship between three xylanases reported in *S. lividans* and *S. viridodiastaticus*. An evolutionary tree was constructed using the neighbor-joining method in the MEGA 6 program. The subjects used to construct the phylogenetic tree included three xylanases from *S. lividans*—xylanase A (XynA_liv_), xylanase B (XynB_liv_), and xylanase C (XynC_liv_)—and three xylanases from *S. viridodiastaticus*—xylanase A (XynA_vir_), xylanase B (XynB_vir_), and xylanase C (XynC_vir_). An evolutionarily distant xylanase (AAB84458.1) from *Bacillus subtilis* was also included in the tree. The evolutionary distance was calculated using the Poisson correction method. Sequence IDs of proteins are indicated in parentheses.

The amino-terminal sequence of the purified xylanase is Ala-Thr-Thr-Ile-Thr-Thr-Asn-Gln-Thr (ATTITTNQT). *In silico* BLASTP analysis at NCBI confirmed that proteins annotated as endo-type xylanases in various *Streptomyces* species had the same sequence as the 9 amino acid sequences mentioned above. Among them, only one open reading frame (GenBank: GHF93985.1), whose 9 amino acid sequences matched perfectly, was found by limiting the scope to the results of the genome analysis of *S. viridodiastaticus*. GHF93985.1 (242 amino acids, 26.1 kDa) is a glycoside hydrolase (GH) family 11 protein [34]. SignalP 6.0 (https://services.healthtech.dtu.dk/services/SignalP-6.0/) analysis predicted that GHF93985.1, had a bacterial twin-arginine translocation (TAT) signal peptide of SRRGFLVG and a cleavage site between Ala-50 and Ala-51. Accordingly, the mature form had a molecular weight of 21.1 kDa (192 amino acids), which is consistent with the molecular weight of the purified enzyme. Additionally, the amino-terminal sequences of the mature forms of the two proteins were identical. Therefore, we concluded that *GHF93985* corresponds to the gene encoding the purified xylanase. *In silico* analysis of GHF93985.1 revealed that it was most similar to xylanase C (XynC_liv_) among several xylanases reported in *S. lividans* (Fig. 5A) and formed a clade in the evolutionary tree (Fig. 5B). Therefore, GHF93985.1 was classified as XynC_vir_ in the sense of *S. viridodiastaticus*-derived xylanase C.

## Discussion

Strain MS9 was isolated as an extracellular xylanase producer from soil sample. The 16S rRNA gene sequence of MS9 showed 100% identity to that of *S. viridodiastaticus* NBRC 13106^T^. Thus, it was named *S. viridodiastaticus* MS9 as a variant of the type strain because of the small differences in DNA–DNA hybridization and chemotaxonomic analysis.

MS9 showed growth under the wide spectrums of pH (pH 5.5-10.0), salt concentration (0-9%, w/v), and temperature (20-50°C), implying it has a high potential to produce heat-/alkali-tolerant enzymes including xylanase. In fact, the extracellular XynC_vir_ purified from the culture broth of MS9 showed endo-type xylanase activity, degrading xylan into various xylooligosaccharides including xylobiose with optimum activity at pH 7.0 and 60°C. Many xylanases from *Streptomyces* species had optimum pH of pH 5.0-7.0 and optimum temperature of 50-65°C [3, 25, 35], and XynC_vir_ also shows the characteristics included in these categories.

Through amino-terminal amino acid sequencing and *in silico* analysis, it was confirmed that the GHF93985.1 protein annotated as a GH11 family protein in *S. viridodiastaticus* corresponded to XynC_vir_. As a result of the BLASTP search at NCBI, hundreds of *Streptomyces*-derived proteins showing very high homology with XynC_vir_ were listed. Among them, the top 100 proteins with the highest homology had an amino acid sequence identity of more than 84.4% with XynC_vir_, and they were annotated as 93 GH11 family proteins, 6 endo-(1,4)-β-xylanase, and one TAT signal domain-containing protein, but none of them were characterized by their biochemical properties.

*S. lividans* is one of the most studied members of the genus *Streptomyces* and several xylanases have been isolated from this strain [27]. Among these, xylanase A (XynA, P26514.2, 477 amino acids), which belongs to the GH10 family, did not show significant homology with XynC_vir_. However, xylanase B (XynB, P26515.3, 335 amino acids) and xylanase C (XynC_liv_, P26220.1, 240 amino acids) belonging to the GH11 family shared 79.2% and 82.7%identity with XynC_vir_ in their amino acid sequence, respectively. Based on the molecular weight (number of amino acids) and % identity, XynC_liv_ in *S. lividans* was estimated to be most similar to XynC_vir_ in *S. viridodiastaticus*. Although biochemical and 3-dimentional studies of XynA and XynB are well established [10, 17], no studies have reported on XynC_liv_. Therefore, our results on XynC_vir_ will be a valuable reference for the functional analysis of many genes encoding proteins with high homology to XynC_vir_ and XynC_liv_.

The amino acid sequences of XynB and XynC_liv_ from *S. lividans* showed 66.1% identity at the 240 amino acid termini. However, the properties of the two enzymes are different in that XynB has an optimum temperature at 40°C and XynC_liv_ at 60°C. According to Wang and Xia [33], these properties depend on the homologous carboxy-terminal regions of each enzyme. In fact, XynC_vir_ shares 79.2% and 82.7% identity with XynB and XynC_liv_, respectively, in its amino acid sequence, and the optimum temperature for the enzymatic reaction of XynC_vir_ is similar to that of XynC_liv_. Moreover, in the carboxy-terminal half region (121-242 amino acids), XynC_vir_ showed 85.0% identity with XynB and 91.8% identity with XynC_liv_. Therefore, it is possible that the optimal reaction temperature of XynC_vir_ is affected by the carboxy-terminal region, similar to that of XynC_liv_.

The bacterial Tat pathway transports folded proteins containing signal peptides with a consensus motif (S/TRRXFLK) across the cytoplasmic membrane. XynC_liv_ contains the SRRGFLG sequence (Fig. 5A) and is secreted via the Tat-dependent secretion pathway, which differs from the secretion of XynA and XynB via the Sec-dependent pathway [12, 21]. SignalP analysis predicted that XynC_liv_ has a cleavage site between Ala-49 and Ala-50, and that the mature form has the same amino-terminal sequence (ATTITTNQT) as XynC_vir_ (Fig. 5A). Based on these results, it can be concluded that XynC_vir_ from *S. viridodiastaticus* is genetically and functionally closest to XynC_liv_ among the xylanases reported in *S. lividans*. XynA and XynB can degrade xylan to produce small amounts of xylooligosaccharides of various lengths and xylose [17, 24]. In contrast, XynC_vir_ breaks down xylan to form xylooligosaccharides, including xylobiose, but not xylose. Therefore, XynC_liv_ seems to be an endo-(1,4)-β-xylanase with enzymatic properties similar to those of XynC_vir_.

Similar to the xylanase reported in *S. lividans*, XynC_vir_ lacks cellulase activity. These properties are necessary for the pulp industry, where only xylan must be removed [28]. We identified candidate proteins WP_093767410.1 (84% identity, 482 amino acids) and WP_266403084.1 (77% identity, 336 amino acids) corresponding to XynA and XynB, respectively, from the genome sequence information of *S. viridodiastaticus* (Fig. 5B). During the purification step of Resource-Q column chromatography, weak xylanase activity was detected in fractions between 16 and 20. These fractions were excluded from the study due to the presence of various protein bands on SDS-PAGE, but it is possible that XynA or XynB candidate proteins of *S. viridodiastaticus* were included in them. Therefore, it is expected that industrially useful cellulase-free xylanases can be developed from *S. viridodiastaticus* through further research on them.
